# Perinatal wellbeing through restoring healthy family systems in a nêhiyaw (Plains Cree) community: a mixed methods study

**DOI:** 10.3389/fpubh.2026.1823280

**Published:** 2026-06-29

**Authors:** Richard T. Oster, Luwana Listener, Denise Young, Marisa Saddleback, Patrick Lightning, Lea Bill, Winnie Chow-Horn, Mandi Gray, Juliette R. Bedard, Rhonda C. Bell

**Affiliations:** 1 Department of Agricultural, Food & Nutritional Science, University of Alberta, Edmonton, AB, Canada; 2 Indigenous Wellness Core, Primary Care Alberta, Edmonton, AB, Canada; 3 Ermineskin Cree Nation, Maskwacîs, AB, Canada; 4 Samson Cree Nation, Maskwacîs, AB, Canada; 5 Alberta First Nations Information Governance Centre, Tsuut'ina, AB, Canada; 6 Department of Sociology, Trent University, Peterborough, ON, Canada; 7 Women and Children's Health Research Institute, University of Alberta, Edmonton, AB, Canada

**Keywords:** Alberta, community-based participatory research, First Nations, Indigenous, maternal health, qualitative research, surveys and questionnaires

## Abstract

**Introduction:**

A collaborative research partnership in Alberta (Canada) was formed with the goal of supporting optimal health during the perinatal period for diverse Indigenous populations. Our approach utilized community-led strategies that promote the best possible healthy life for families and children. This partnership was guided by a Restoring and Supporting Healthy Family Systems (RSHFS) conceptual model based primarily on Indigenous ways of knowing. We sought to explore components of the RSHFS model with members of a nêhiyaw (Plains Cree) community of childbearing age, focused on refining codesigned perinatal strategies and supports for women and families.

**Methods:**

We utilized a convergent parallel mixed methods design. In collaboration with nêhiyaw community members, we developed and implemented a survey for women of childbearing age and their partners. Follow-up semistructured interviews were carried out. Data generation approaches were guided by the RSHFS model that incorporates seven interrelated elements: identity, supportive environments, culture, language, grandfathers and grandmothers, fathers and mothers, and extended family.

**Results:**

Fifty-two participants completed the survey and seven completed the interviews. All seven domains of the RSHFS model were found to be salient and interconnected influences on perinatal wellbeing. Participants emphasized the importance of supportive family relationships and connections to culture, language, identity, and land, while also describing the ongoing impacts of intergenerational trauma, gaps in perinatal supports, and culturally unsafe health care interactions.

**Discussion:**

Perinatal wellbeing in this nêhiyaw community is influenced by interconnected family, cultural, and structural systems. The RSHFS model reflected participants' lived experiences and identified pathways to restoring family relationships, strengthening cultural identity, supporting healing, providing additional perinatal supports and system navigation, and addressing cultural safety.

## 1 Introduction

Until relatively recently, much of the literature on Indigenous health has been from a western scientific lens, which most often relies on a deficits-based perspective (1, 2). Health disparities between non-Indigenous and Indigenous people (First Nations, Inuit, and Métis) across Canada are well documented and are more pronounced for Indigenous women during the perinatal period (3–8). While the literature is important for understanding health care inequities that disproportionately impact Indigenous women, it tends to exclude evidence of the resiliency of Indigenous Peoples and their communities, potentially perpetuating harmful stereotypes and exacerbating marginalization within the health care system (2, 9).

Strengths-based and community-appropriate approaches rooted in Indigenous cultural practices are needed to improve perinatal wellbeing in Indigenous communities (10–14). In 2019, partners from various research disciplines and communities came together through community-based participatory research (CBPR) as part of the Indigenous Healthy Life Trajectories Initiative (I-HeLTI) to enhance preconception and perinatal (pregnancy to approximately 1 year postpartum) health for diverse Indigenous populations in the province of Alberta, Canada. Specifically, our team aims to incorporate Indigenous knowledge, wise practices, and cultural teachings into appropriately designed and effectively evaluated strategies that help Indigenous communities move toward rebuilding family and community systems, with positive impacts on the health of pregnant Indigenous mothers, newborns, their families, and communities.

Central to our partnership is the development and use of the working conceptual model Restoring and Supporting Healthy Family Systems (RSHFS). This model was created by co-author and Knowledge Holder LB early in our collaboration, grounded in traditional Indigenous principles of a healthy family as a whole system. In this project, we explored components of the RSHFS model with community members of childbearing age of a partnering I-HeLTI nêhiyaw (Plains Cree) community. It is one part of a process aimed at refining and strengthening existing perinatal support strategies for women and families that have been codeveloped.

## 2 Materials and methods

### 2.1 Research partnership

The Alberta I-HeLTI team represents a collaborative research relationship between Indigenous communities and academic researchers, bringing together diverse Elders, Knowledge Holders, community leaders, Indigenous and non-Indigenous scholars, and health care providers from across the province. The present study emerged from a long-term partnership between researchers at the University of Alberta and members from the nêhiyaw community of Maskwacîs that began in 2013, uniting as the Wâhkôhtowin Research Group (WRG). Maskwacîs is a hub for nêhiyaw culture, located in rural Treaty 6 Territory, and includes four distinct but interrelated First Nations: Samson Cree Nation, Ermineskin Cree Nation, Louis Bull Tribe, and Montana First Nation. The WRG is led by a core team of community researchers from Maskwacîs and is based on the guiding principle of wâhkôhtowin—we are all related; therefore, trusting and genuine relationships are foundational. This study and the WRG are informed by the principles of CBPR and an understanding that community members are experts, best positioned to know their own context and to hold the knowledge necessary for research that will benefit their community (15, 16). Over many years of collaborative research, the WRG has codeveloped support strategies for women and families that are ongoing, community-led, and include embedded research components. Two of these support strategies have been in place for several years and serve as interventions for the Alberta I-HeLTI program. The Moms Support and Healing Circle, which evolved from an Elders Mentoring Program (11, 14), provides consistent opportunities for moms and moms-to-be to gather in community to connect in a culturally rooted environment. Similarly, the Deadly Dads Support Society (17, 18), offers ongoing opportunities to foster a sense of belonging, support, and knowledge-sharing for fathers, fathers-to-be, and men in the community.

The current study was guided by a community advisory committee (CAC), which included community members, Elders, Knowledge Keepers, and university researchers. The CAC was involved in all stages of the project and ensured that the research was conducted in a way that was culturally appropriate and that would benefit the community. The partnership is constantly evolving and has been described in previous publications (14, 17, 18). The research team for this study consisted of community researchers from Maskwacîs, a nêhiyaw Knowledge Holder and scholar, and longtime non-Indigenous academic research partners. Our positionality reflects our commitment to working together in a good way, leading with wâhkôhtowin. Team members' individual positionality statements are available in the Supplementary material.

### 2.2 Restoring and supporting healthy family systems model

**Figure 1:**
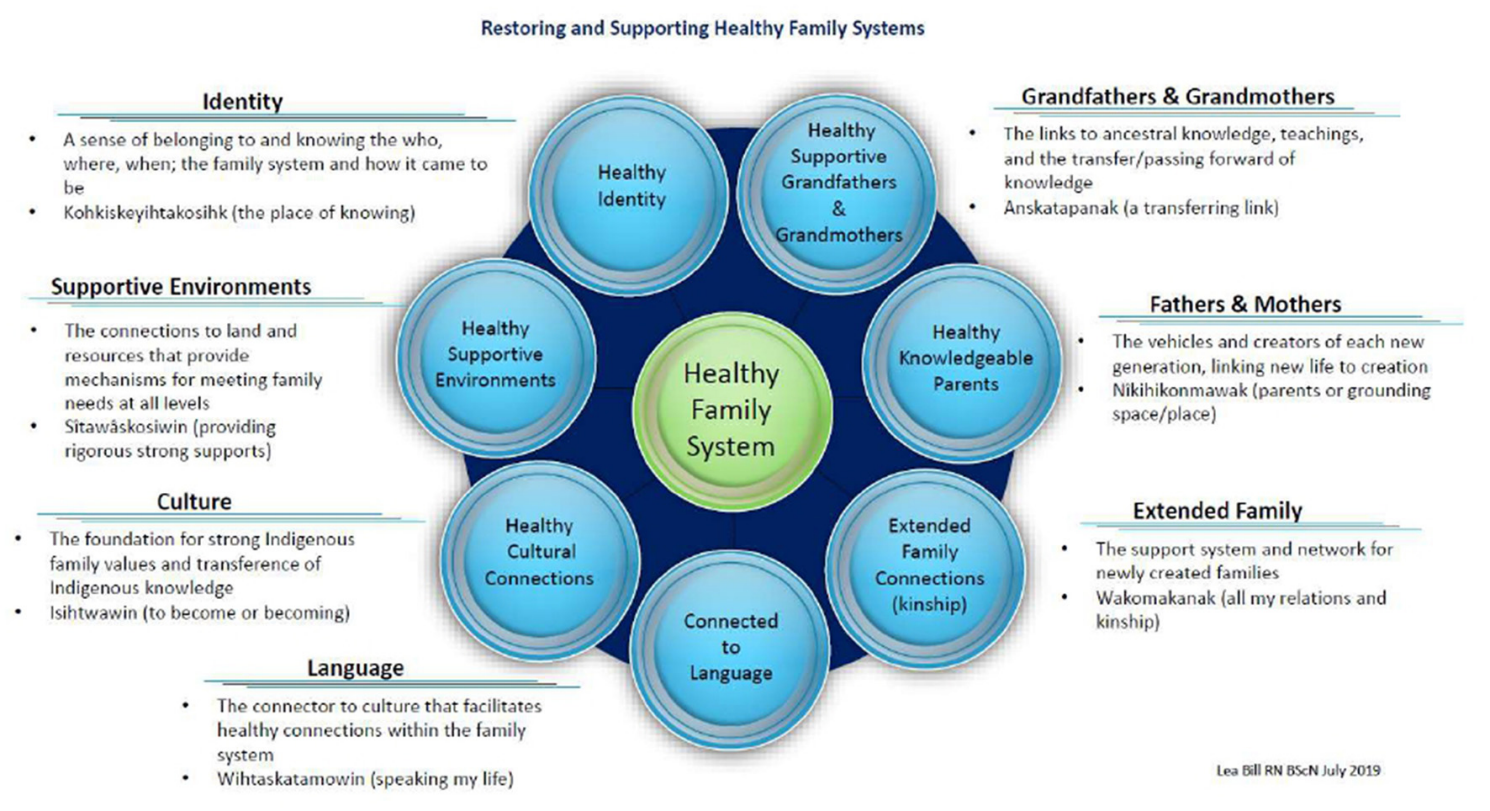
Restoring and supporting healthy family systems framework.

Knowledge Holder and co-author LB defines Indigenous family as residing in a worldview in which “we are all related”—a perspective that transcends the notion of family as a self-contained unit independent of connections to the natural world. Accordingly, the RSHFS model (Figure 1) includes seven circles of knowledge: Identity, Kohkiskeyihtakosihk (place of knowing); Supportive Environments, Sîtawâskosiwin (providing rigorous, strong supports); Culture, Isihtwawin (to become or becoming); Language, Wihtaskatamowin (speaking my life); Grandfathers and Grandmothers, Anskatapanak (a transferring link); Fathers and Mothers, Nîkihikonmawak (parents or grounding place/place); and Extended Family, Wakomakanak (all my relations and kinship). Grandfathers and grandmothers play a central role in passing down ancestral teachings, helping family members understand where they come from and how they are connected. Supportive environments rooted in healthy identity, cultural continuity, and shared knowledge create the foundation for wellness across generations. Fathers and mothers connect the family to the land and resources, ensuring physical, emotional, and cultural needs are met and providing the grounding force for new generations. The extended family of all relations strengthens kinship ties, cultural values, and the transfer of Indigenous knowledge. All relations form the essential support network for new and growing families. Language, a key connector to culture, supports healthy relationships and identity within the whole family system. Overall, a healthy family system emerges when identity, culture, supportive environments, knowledgeable parents, and strong kinship ties work together to nurture both current and future generations. From an Indigenous perspective, the background circle encompassing all seven circles of knowledge represents the amniotic waters in which all beings are contained, supported, and nurtured by the intrauterine experience. Each of the circles resting on the sacred waters emerges as a bubble, and key bodies of knowledge, teachings, and skills are required to achieve health within a family system.

Given the overarching RSHFS model for our I-HeLTI collaboration, we aimed to explore each domain with community members of childbearing age to ensure the concepts resonated and to provide direction for refining our existing support strategies for women and families.

### 2.3 Study design

We used a convergent parallel mixed methods design that included a survey and follow-up qualitative interviews (19). This design leveraged the strengths of both data sources by collecting and analyzing quantitative and qualitative strands separately within the same research phase, then integrating and interpreting the results together.

### 2.4 Data generation and analysis

The CAC prioritized developing indicators of wellbeing that were distinctions-based and relevant to Maskwacîs and its people. Thus, data generation approaches were developed through a series of discussions led by Naheyawin, a local Indigenous consulting group, in collaboration with the CAC and members of the WRG. Discussions centered on interpreting the RSHFS model to best reflect the communities of Maskwacîs.

The survey included 22 questions specific to the RSHFS model and worded from a strengths-based perspective (see Table 1). The survey was pilot-tested with a small subgroup of community members, and their feedback was incorporated into the final drafts distributed in the community. Discussions with the CAC also provided insights into best practices for engaging community members in the research process, including how to encourage research participation and ensure informed consent. Survey participants received a $25 gift card as a token of appreciation.

**Table 1:** Restoring and supporting healthy family systems model domain survey responses (*n* = 52).

Restoring and supporting healthy family systems model domain	Question	Disagree or strongly disagree	Neutral	Agree or strongly agree	Missing
		No. (%) of participant responses			
Healthy supportive grandfathers and grandmothers	I regularly visit with my/my children's grandparents (*n* = 51)	14 (27)	13 (25)	24 (46)	1 (2)
Healthy supportive grandfathers and grandmothers	I feel connected to the past through stories, teachings, and knowledge shared by my/my children's grandparents (*n* = 51)	3 (6)	17 (32)	31 (60)	1 (2)
Healthy supportive grandfathers and grandmothers	My/my children's grandparents are trying to become healthier, more supportive family members (*n* = 51)	5 (10)	9 (17)	37 (71)	1 (2)
Healthy knowledgeable parents	My parents have a significant role in my life today (*n* = 51)	5 (10)	14 (27)	32 (61)	1 (2)
Healthy knowledgeable parents	My parents are trying to become healthier more supportive family members (*n* = 50)	6 (11)	12 (23)	32 (61)	2 (5)
Healthy knowledgeable parents	I have a significant positive role in the lives of my children (*n* = 45)	1 (2)	10 (19)	34 (66)	7 (13)
Healthy knowledgeable parents	I am trying to become a healthier, more supportive parent to my children (*n* = 45)	1 (2)	10 (19)	34 (66)	7 (13)
Extended family connections	I regularly visit with my/my children's extended family members (*n* = 51)	11 (21)	12 (23)	28 (54)	1 (2)
Extended family connections	I feel connected to the past through the stories, teachings, and knowledge shared by my/my children's extended family (*n* = 50)	8 (15)	12 (23)	30 (58)	2 (5)
Extended family connections	Those in my extended family are trying to become healthier and more supportive (*n* = 49)	5 (10)	12 (23)	32 (61)	3 (6)
Connected to language	Language is important to my family and I (*n* = 50)	0 (0)	3 (6)	47 (90)	2 (4)
Connected to language	I can find resources and support for learning and speaking my language that fit my busy life (*n* = 50)	7 (13)	10 (19)	33 (64)	2 (4)
Connected to language	I have tried to learn more about my language, I am proud of the progress I am making (*n* = 50)	5 (10)	17 (32)	28 (54)	2 (4)
Healthy cultural connections	I understand what Indigenous family values are (*n* = 50)	0 (0)	7 (13)	43 (83)	2 (4)
Healthy cultural connections	Strong values are key to a healthy family (*n* = 50)	0 (0)	4 (8)	46 (88)	2 (4)
Healthy cultural connections	I make every effort to lead my family by example by living in line with values that are important to me (*n* = 50)	2 (4)	6 (11)	42 (81)	2 (4)
Healthy supportive environments	My family has a safe place to live and grow together (*n* = 50)	2 (4)	6 (12)	42 (80)	2 (4)
Healthy supportive environments	Connection to the land is important to my family (*n* = 50)	0 (0)	5 (10)	45 (86)	2 (4)
Healthy supportive environments	I can find resources and support for building and maintaining my connection to the land (*n* = 50)	1 (2)	14 (27)	35 (67)	2 (4)
Healthy identity	I am important and I have much to offer my family and community (*n* = 50)	2 (4)	5 (10)	43 (82)	2 (4)
Healthy identity	The future holds positive, exciting things for my family and I (*n* = 50)	0 (0)	4 (6)	46 (88)	2 (4)
Healthy identity	My ancestors would be proud of me (*n* = 50)	2 (4)	7 (13)	41 (79)	2 (4)

The CAC deliberately advised for open inclusion criteria to honor self-determination and inclusivity in our approach, ensuring that no one was intentionally excluded from sharing their voice. Consequently, all women of childbearing age and their partners were invited to complete the survey. The research team used a variety of recruitment methods, utilizing primarily convenience sampling and incorporating elements of self-selection sampling. Taking direction from our CAC, this sampling approach prioritized relationships and participant safety over statistical representativeness. At the start of the project, we hosted a community lunch to share information about the project and recruit survey participants. Additional participants were recruited at a community powwow. The survey was also promoted on social media and through community posters inviting participation. Community researchers from the WRG facilitated deployment of the surveys either in-person or over the phone. Completed paper surveys were converted into electronic format. Survey data was collected from July to September 2021. In line with our relational and community-led approach, we did not have a target sample size. Instead, we sought to include all community members who felt moved to share their perspectives, ensuring that the final data reflected community interest and readiness. All survey responses were uploaded into a REDCap database hosted at the University of Alberta. Data was cleaned and analyzed using Tableau Prep and Tableau. Microsoft Excel was used as a tool for quality checking. We maintained a purely descriptive approach to analysis to ensure the findings remained accessible and actionable for community-led advocacy. As advised by the CAC, this method prioritizes community data sovereignty by focusing on the internal strengths and lived realities of participants rather than using inferential models that can lead to deficit-based comparisons.

Survey participants had the opportunity to participate in a one-on-one, qualitative, semistructured interview. All those who expressed interest were interviewed. Interview discussions were guided by broad topic areas related to the RSHFS domains, including family relationships, parenting, culture and language, identity, community supports, and experiences accessing services. Interviews remained conversational and participant-led, allowing individuals to emphasize topics most relevant to their experiences. Participants were also asked about their experience with Indigenous-led and western health care services (not reported here). Interview participants received a $50 gift card as a token of appreciation.

WRG community researchers conducted interviews from February to April 2022. Interviews were recorded and transcribed using Otter.ai, while NVivo was used for thematic coding and analysis. Qualitative data generation and analysis occurred concurrently. The seven domains of the RSHFS model were used as a coding framework to analyze the interview transcripts. Community researchers analyzed the transcripts, as their community experience and knowledge provided a deep understanding of the data. The coding and theming process was conducted through five iterative rounds: (1) general coding of transcriptions; (2) identifying emerging themes, salient quotes, and interpretations of gaps in services and supports for healthy pregnancies and experiences within the health care system; (3) using the RSHFS model to assess identified themes in comparison to the framework's seven domains for alignment, as well as new emerging themes and interpretations; (4) reviewing interpretations to find saturation of themes within the framework, forming the basis of consensus for main strategies identified by participants; and (5) carrying out a comparative analysis of the main strategies, identifying key gaps and barriers in services, to determine priority strategies that would have a high impact on perinatal wellbeing. Data saturation was reached when no new information or insight emerged, and when the categories were well defined. At the conclusion of the analysis, the findings were presented to and discussed with the CAC. This process resulted in further refinement and verification of the findings, reducing redundancy and ensuring accuracy and community appropriateness. The WRG and CAC identified core principles specific to conducting research in a good way with Maskwacîs: trust, strong relationships, mutual benefit, equitable partnerships, knowledge-sharing, the incorporation of cultural teachings, and action-oriented and strengths-based research. These principles were not only important for ensuring the rigor and credibility of the qualitative findings, but they also reflected relational and reciprocal approaches to research that were important to community members and central to conducting research in a respectful and culturally grounded way in Maskwacîs.

Quantitative and qualitative data integration occurred at the reporting level through a weaving approach (20). By integrating findings through narrative integration, we presented survey findings and qualitative insights side-by-side. This allowed the qualitative narratives to provide the necessary cultural depth and nuance to explain the context behind the quantitative trends.

### 2.5 Ethics

This study received ethics approval from the University of Alberta Research Ethics Board (Pro0009113), which adheres to the “Tri-Council Policy Statement 2, Chapter 9: Research Involving the First Nations, Inuit, and Métis Peoples of Canada” (21). The research team also obtained community approval for this project from the CAC, who were approached using community-appropriate protocol. All study participants provided written informed consent. Also, prior to data generation, a research agreement was collaboratively established by the CAC, the Chief Executive Officer of Maskwacîs Health Services (a long-standing partner with WRG) and partnered academic researchers. Control over research is situated within the community, and the research agreement outlines that all generated data is owned, controlled, accessed, and possessed by the WRG. As such, data is stored long-term within the community on Maskwacîs Health Services secure servers, with access restricted to the WRG.

## 3 Results

A total of 52 participants completed the survey; 73.1% identified as women. The survey participants ranged in age from 16 to 50 years, with a mean age of 32.7. Most participants (70.0%) were already parents, and 4.0% were either pregnant or had a pregnant partner. Another 26.0% were not parents, and among this group, 75.0% indicated they planned to become parents someday. The majority of respondents (84.6%) lived in Maskwacîs, while 15.4% lived in nearby communities.

Seven survey respondents also participated in one-on-one qualitative interviews. Six identified as women and one as a man. Interview participants ranged in age from 23 to 43 years.

Results of the survey questions are presented in Table 1 and described in relation to the following qualitative findings. Supportive illustrative qualitative quotations are provided in Table 2. Collectively across the domains, survey findings consistently demonstrated high endorsement of the importance of culture, identity, family relationships, and connection to land. However, qualitative interviews revealed substantial challenges in accessing or sustaining these supports due to intergenerational trauma, disrupted family systems, racism, violence, and structural barriers. Together, the findings suggest that participants viewed perinatal wellbeing not simply as an individual responsibility, but as deeply shaped by relational, cultural, and environmental conditions.

**Table 2:** Supporting quotations from interview participants.

Restoring and supporting healthy family systems model domain	Illustrative quotations
Healthy supportive grandfathers and grandmothers	“Just talking with an Elder is so powerful and meaningful. Because you feel this sort of protection, you know? And it makes you feel safe; it makes you feel heard, even if you can't see it. It's not physically there, but you feel it”
	“I would've um talked to more Elders… That was a big thing I missed out on… And I realize how important it is to have a healthy relationship with your grandmother because I didn't have that. Somebody you could go to, that could give you good advice and still give you shit when you need it. You know, and [someone who] won't encourage bad behavior”
	“I feel really blessed my daughter can grow up to have her grandmother because we had grandparents, but they were active in abuse in the family”
Healthy knowledgeable parents	“It is very important to have healthy pregnancies. And then nowadays you have a lot of our women that drink while they're pregnant. And that right there is a crime…. That's why I'm here, because I grew up with both parents… I felt safe with my mom and dad”
	“I was very fortunate that I always had my parents there. With all my pregnancies, I was very lucky that my mom really pushed the same thing, you know, that exercise and walking. It's always the walking, you know, you don't have to go lift weights, you don't have to go swimming a marathon and all that shit, as long as you‘re doing the physical walking yourself. And that's telling your body that, hey, we're preparing for a big ceremony”
	“Being a product of the residential era, being a part of the genocide, I grew up with the drugs and alcohol in home. And where did it get me? Not very far. If I had a better understanding of it, and if I had more of a chance to know better at a younger age, you bet I would have made those healthier choices rather than continuing to live in hell… I wasn't educated at all in any way… So, no one set us up to say, ‘when you grow up, you know, you got to practice safe sex, you got to get an education, if you want those kick ass jobs.' So, there's nobody telling you in your youth that growing up is a big part of life. Instead, you're kept in a dark thinking, you're going to be a kid forever, a child forever,
	somebody's responsibility. And then when you are forced to go get your own responsibility, you don't even have a clue how to figure that out, you don't even know how to pay bills, you don't know how to get your taxes done. There's so many things that are kept from our children. Because of the things that happen in residential, there's lots of lost teachings… It was just like no, do what you want, live how you want, and make a mess. So that's what I did. I made a mess of my life. And then when I finally realized what a mess it was, I realized it's possible to clean it up. And it has to come from within. No one can force you to change, you have to want it so deep and so bad that you're willing to look at the truth for what it is, no matter how ugly it is”
	“My main goal is to raise my daughter in a healthy, happy environment so she doesn't have a childhood [she'll need] to heal from… Her father is not involved. He was incarcerated when she turned 5 months. And when he was incarcerated, I was approached by numerous people, family and friends, that he had molested and raped family members, young members. And having my own traumas, that's my biggest fear in life. And for my own safety, [and my daughter's] safety, he's not in our life”
Extended family connections	“So, it's nice to have my daughter to have that bond, she's very close with my sister as well… Her auntie is her best friend, her everything… That's something I'm very grateful for is strong family dynamics, my mom and my sisters… They have a very, very close bond, but due to the abuse in the family my daughter really only knows my mom, my two sisters and my niece. The extended family is obsolete she doesn't know them”
	“That's how it is with my relatives, my family, it just keeps repeating… Because even those abusers are done by cousins, [they] went on to abuse other kids and then their own children… Because this happens too often, and it's usually by family members or people that they know. And that's what is so sad is that they teach you that it's some stranger, they teach you that, oh, don't talk to strangers, because this could happen. In reality, it's someone you know”
	“I'd say it's still broken, like the dynamic of that, of that family unit. It's, it's not there. In most of our families, we are no longer serving each other, and trying to help each other to grow and excel… And it's like, what happened to sharing? What happened to sharing all the stuff together as a family?”
	“It's hard to trust because a lot of times they've [extended family members] hurt us or perpetuated it because of intergenerational trauma. It cascades down to generations, and hurt people just hurt people. And we have to define families in a new way, by just making our support group by who we trust and who we were friends with, not only from families”
Connected to language, healthy cultural connections, and healthy identity	“If we were, more people were to, you know, engage in our cultural ways it would do tremendous amounts for them… Because we can keep everything inside us to hide it, but we can't hide it from the Creator or our Grandfathers in the sweat”
	“Culture is a way of life. It's, it's something that you do every day, you know, it's just a part of you. It's like, it's there. And even if you feel like it's not, it is, it's a part of you. Like, you may not practice it every day, but it's still there… So, to me, it's something that connects our people a lot in a place that feels very disconnected. And it can do a lot of healing”
	“Without culture, we got nothing to stand on because there's nothing behind us supporting us. It's like that backbone, that spine that you have, you need something to support that and that is that root. The culture brings you to your roots. It keeps you tied to that root. So that way when you go out into the world, you know where your roots come from”
	“Somewhere along the line, our culture has taken a backseat. So, I think that the kids need to come back to the culture to really know and realize their true selves. They're not these little want-to-be gangbangers, shitty little drug dealers, and all that that's not who we are. But they don't have anyone to teach them to deal with this intergenerational shit. So, we have to break that. Culture has [to have] more access I guess you could say for these kids. Then we can turn it around and be who we‘re really supposed to be”
	“I'm carrying on the ways of our people. These ceremonies, they are getting lost, and they're not being done, right. So, if we learn how to be done, properly, teach the kids and then they can teach their kids for generations to come. You know, there can be a deterrent [against loss of identity]”
Healthy supportive environments	“You need love, support. You need a good support system. They should have support groups for other young moms, where they can talk to other young moms, you know, let them know in a fun way and a social way... To get that help without being embarrassed and or feeling like they're [asking a] stupid question, you know”
	“The medical system is really hard to deal with, especially if you're Aboriginal… The nurses there, some of them are really, really rude. They're very mean and because of all my conditions, they always assume immediately that I'm OD'ing. [They assume] it's because I OD'ed or I drink too much. And like I said I've been sober now for it'll be 8 years. And I don't do drugs, so. Before I get care, they'll do a toxicology report immediately and just tell whoever is on call or the doctors that I'm just there for pain meds. So then my doctor had to write a letter for me, that's what I give hospital at the time I go, wherever I go it lists all my conditions, all the symptoms, all the medications I'm on, that I don't do drugs, that I don't drink and he signed it off that way they cannot do that to me at the hospitals anymore”
	“[There are] real scary, and bad and evil kind of things. Because the way people live, like really messy houses and uh lots of drugs and alcohol around. The loose dogs that are all over outside. No one feels completely safe… [There are] no boundaries in my ghetto… But now that they're trying to clean up the town side, you feel a bit more safer when they're really pushing to put family families in these units. Not partiers”

### 3.1 Healthy supportive grandfathers and grandmothers

From the survey, 60.8% of participants agreed or strongly agreed that they felt connected to the past through stories, teachings, and knowledge shared by their grandparents or their children's grandparents. The importance of having healthy grandparents involved in their own and their children's lives was mentioned by all interview participants as crucial to providing culture, ceremony, language, teachings, and a sense of protection and safety.

Several interview participants expressed feeling blessed that their own children had healthy grandparents in their lives, and 47.1% of survey participants agreed or strongly agreed when asked if they regularly visited grandparents. Some interview participants longed for healthy grandparents while growing up, did not have grandparents present, and, in some cases, experienced abuse from grandparents.

Interview participants shared that many grandparents in the community either needed to be, or were actively on, a healing journey. When asked if their children's grandparents were working on becoming healthier, more supportive family members, 72.6% of survey participants agreed or strongly agreed. Interview participants discussed grandparents as a means of reconnecting to culture toward healing their own traumas as important for repairing and supporting healthy families into the future.

Taken together, these findings suggest that grandparents were viewed simultaneously as sources of cultural continuity, healing, and safety, but also sometimes as individuals impacted by unresolved trauma and disrupted family relationships.

### 3.2 Healthy knowledgeable parents

Most survey participants (75.6%) agreed or strongly agreed that they had a significant, positive role in the lives of their children. Some interview participants credited this to having parents who were or are healthy role models during their youth. Like healthy grandparents, interview participants emphasized the importance of having a healthy pregnancy and being a healthy parent as central to raising children well. Participants explained that raising children well requires maintaining and modeling a healthy lifestyle, including nutritious eating, regular exercise, abstaining from substances, supporting mental and emotional wellbeing, ensuring a safe home environment, staying connected to culture and ceremony, and being actively present in their children's lives.

When asked whether they were working on becoming healthier and more supportive parents to their children, 75.6% of survey participants agreed or strongly agreed. Some interview participants connected being a healthy parent to having knowledge of the impacts of colonization and intergenerational trauma, as well as taking personal responsibility and accountability for improving one's life. They also noted that healthy parenting was closely tied to having a supportive, healthy partner and maintaining strong, healthy partner relationships. Moreover, they described how unhealthy habits of either parent directly and negatively impact the health of babies, children, families, and communities. Many interview participants noted trying to break cycles of abuse and shield their children from the unhealthy habits of their partners.

A total of 62.7% of survey participants agreed or strongly agreed that their own parents have a significant role in their lives today. However, several interview participants shared stories of immense difficulties, such as being emotionally disconnected from parents who were suffering from abuse and/or addiction, which created repeating patterns of pain, emptiness, trauma, and loss of teachings. Some interview participants described that their parents were taking positive steps toward their own healing, whereas others did not. Among survey participants, 64.0% agreed or strongly agreed that their parents are working on becoming healthier, more supportive family members.

Although participants strongly endorsed the importance of healthy parenting practices, qualitative narratives highlighted how parenting experiences were often shaped by unresolved trauma, substance use, disrupted teachings, and unstable partner relationships. The qualitative interviews revealed a strong desire among participants to repair and restore connections with their parents that were ruptured due to the ongoing impacts of colonization.

### 3.3 Extended family connections

Among survey participants, 60.0% agreed or strongly agreed that they felt connected to the past through the stories, teachings, and knowledge shared by their extended family, and 54.9% agreed or strongly agreed that they regularly visited extended family. Interview participants frequently spoke of extended healthy families as critical to the wellbeing of babies, families, and communities. Some interview participants reported healthy family connections that resulted in positive, stable, and happy households, whereas others shared disconnected and broken families that led to isolated, unhealthy individuals and families.

All interview participants explained having family members who survived residential schools. When asked if they felt that those in their extended family were working on becoming healthier and more supportive, 65.3% of survey respondents agreed or strongly agreed. Some interview participants described cycles of repeated dysfunction, violence, neglect, and abuse within extended families, highlighting the need for healing from intergenerational trauma.

Interview participants conveyed how extended family members do not have to be blood relations; they can be friends, support groups, or others in the community where there is established trust. Several interview participants expressed a desire to connect with other supporters (e.g., other mothers) within the community.

These findings highlight the complex role of extended family as both a source of support and connection and a site of intergenerational trauma, underscoring the importance of rebuilding trusted support networks within and beyond biological family structures.

### 3.4 Connected to language, healthy cultural connections, and healthy identity

The RSHFS model domains labeled Connected to Language, Healthy Cultural Connections, and Healthy Identity are combined here, as participants routinely articulated these constructs as inextricably linked. Survey participants consistently reported strong endorsement of language, and understanding cultural values, strong values as key to a healthy family, and living in line with values, with agreement levels ranging from 84 to 94%. Interview participants described their community as immensely rich in culture and language, which one participant referred to as the “roots of existence” and as foundational to a good way of life, wellbeing, identity, balance, spiritual connection, and peace. Culture and language were frequently mentioned as facilitators of good health, healthy families and communities, and a means of healing and connecting families.

However, several interview participants stated that for many within the community, the connection to culture and language has been severely disrupted. They expressed that many ceremonies are being lost, or as one participant noted, “contaminated by the western world” (e.g., due to a lack of Cree language and the inclusion of unhealthy, non-traditional foods), leading to loss of identity and, ultimately, poor health among families. Interview participants explained how colonization has severed the connection to culture and that deep healing within the community is needed. They further illustrated a yearning within the community to learn about and engage in cultural activities, including access to ceremony, traditional pregnancy and parenting teachings, and traditional foods through land-based teachings. Approximately two thirds (66.0%) of survey respondents agreed or strongly agreed that they can find resources and support for learning and speaking their language, while 56.0% agreed or strongly agreed that they have tried to learn more about their language and are proud of the progress they are making.

Most survey respondents agreed or strongly agreed that they felt they were important and have much to offer their family and community (86.0%); that the future holds positive, exciting things for them and their family (92.0%); and that their ancestors would be proud of them (82.0%). Many interview participants described their culture as fundamental to their identity. However, several interview participants expressed that many youth in the community are lost, do not know where they fit in, lack purpose, and are missing identity. They shared that these young people are not engaging in cultural activities or ceremonies, learning their language, praying, or embracing their Indigenous identity. Loss of identity was also mentioned as common across all age groups and linked to ongoing cycles of intergenerational trauma and abuse. Participants identified connections to language, culture, spirituality, and their ancestors as the path to forming healthy identities.

Notably, although participants overwhelmingly validated the importance of culture, language, values, and identity, interviews revealed concerns about erosion of these practices and unequal access to cultural teachings and supports. This suggests that cultural continuity was experienced both as a major strength within the community and as an area requiring ongoing restoration and investment.

### 3.5 Healthy supportive environments

A total of 90.0% of survey respondents agreed or strongly agreed that connection to the land was important to their family, and 70.0% agreed or strongly agreed that they could find the resources and support necessary for building and maintaining connection to the land. Moreover, interview participants regularly mentioned a desire to talk with other women or mothers for support. For interview participants, health care was seen as key to a healthy, supportive environment. They provided critical insight into the health care system as it currently exists, as well as possibilities for improving care, such as several existing programs that were helpful to them, including parenting classes and nutrition support, although transportation was often a barrier to access. Yet many expressed feeling unaware of and disconnected from prenatal programs and services in the community. Some highlighted the need for improved navigation systems within the community. Another suggestion was the need for improved interagency collaboration to provide wraparound support for families and the ability to share resources among agencies serving the population. Interview participants expressed a desire for additional support in surviving sexual abuse, grief and loss, midwifery and perinatal care, and cultural and language programs with Elders; these supports should also be land-based.

Some interview participants identified excellent relationships and interactions with health care providers that were supportive, helpful, easy, accessible, attentive, and relational. However, others did not have the same experience, noting difficulties accessing health care (e.g., transportation issues, long wait times), quick visits that were impersonal, and restrictive inflexible clinic policies. Participants noted the need to improve the availability and access to culturally responsive and trauma-informed health care professionals in their community and surrounding areas. Some interview participants detailed negative interactions with health care providers that they felt were racist, rude, impersonal, and predicated on negative stereotypes about Indigenous people. This resulted in feelings of hopelessness, anger, and frustration, and ultimately had a negative impact on their wellbeing.

Among survey respondents, 84.0% agreed or strongly agreed that their family had a safe place to live and grow. However, unsafe environments were also frequently outlined, where substance abuse, addiction, crime, violence, sexual assault, grief, partiers, gangs, trauma, as well as judgment and gossip by neighbors negatively impacted their sense of safety and feeling of support. When discussing healthy supportive environments, interview participants mentioned the importance of their neighbors. Some expressed having neighbors who were healthy, and in turn, participants felt safe and as though community members were looking out for one another.

These findings suggest that while participants highly valued supportive environments and connection to land, experiences of racism, unsafe community conditions, and difficulty navigating services often undermined feelings of safety and support.

### 3.6 Summary and application of the findings to support strategies

Collectively, the findings highlighted that strengthening perinatal wellbeing in Maskwacîs requires approaches that simultaneously support cultural continuity, family healing, relational support, and culturally safe systems of care. Participants highlighted culture, language, family relationships, and connection to land as central strengths that promote wellness, identity, and healing. At the same time, they identified ongoing challenges associated with intergenerational trauma, disrupted family relationships, racism, violence, and limited access to culturally grounded supports and services. Together, these findings suggest that perinatal wellbeing is deeply shaped by relational, cultural, and structural contexts.

After the data analysis was completed, a series of conversations within the WRG and the CAC ensued, aiming to incorporate findings into the evolving codesigned perinatal support strategies (the Moms Support and Healing Circle and the Deadly Dads Support Society) so that they better align with the needs of community members and the RSHFS model. Based on the findings, several key areas for investment to refine the existing strategies were identified:

- **Repair and heal relationships in the family system that have been ruptured by intergenerational trauma and colonization**. Accordingly, codesigned strategies and our entire research group seek to be as inclusive as possible, accepting all community members who wish to join. Recent and planned sessions for strategies focus on encouraging healthy interpersonal relationships, making connections with healthy family members, increasing knowledge of intergenerational trauma and healing, and breaking cycles of abuse within families.
- **Further enhance connections to culture and language for youth and families**. By embedding cultural, language, and ceremonial activities into every session, the codesigned strategies prioritize traditional knowledge and land-based teachings. This approach fosters a strong sense of identity and belonging, establishing the purpose, pride, and self-worth necessary for participants to live, survive, and thrive.
- **Provide additional perinatal support and system navigation**. Codesigned programs center their sessions on providing opportunities to create and enhance new social support networks with peers. Activities underway or being planned address topics such as surviving sexual abuse, grief and loss, and healing trauma. Additionally, a “community guide” perinatal service navigation tool has been developed to connect families to medical, social, mental, emotional, physical, and spiritual resources that they may need.
- **Address cultural safety in clinics and services that support families during the perinatal period**. Codesigned strategies aim to provide culturally responsive, trauma-informed support within a safe and non-judgmental community-based environment, addressing a significant gap in mainstream health care services. The strategies have also involved planning and carrying out a variety of engagement activities to build relationships and cross-cultural understanding, including with non-Indigenous health care workers.

## 4 Discussion

This mixed methods study contributes to the growing literature emphasizing Indigenous perinatal wellbeing is shaped by relational, cultural, and structural factors rather than individual behaviors alone. We explored perinatal wellbeing through the RSHFS model within the context of Maskwacîs and the lived experiences of community members of childbearing age. Participants described each of the seven domains of the RSHFS model as deeply relevant to their health and wellbeing as individuals, families, and as a community. Participants' experiences highlighted how these domains work together to support healthy families. As our CBPR partnership advances, the findings provide tangible directions for refining existing perinatal support strategies.

Until relatively recently, most studies on Indigenous health focused on individual and community deficits. Our findings reinforce the value of strengths-based and CBPR approaches, which position community members as knowledge holders and co-creators of solutions (15, 16). This study demonstrates that community-derived models, such as RSHFS, can guide meaningful data generation while directly informing program refinement and service delivery. The strong resonance of the RSHFS domains suggests that Indigenous conceptual models offer an effective alternative to western biomedical frameworks, which often fragment perinatal care from family and cultural systems.

Our study also demonstrates how the RSHFS model moves beyond standard social determinants of health frameworks by positioning family healing and cultural resurgence as central drivers of perinatal wellness. In doing so, it offers a community-validated alternative to deficit-oriented approaches that have historically fragmented care from wider kinship structures. This has important implications for health education and promotion, as it suggests that culturally safe, relational, and land- or community-based approaches may be more meaningful and effective than conventional educational models focused primarily on individual risk reduction.

Indigenous scholars have long argued that western biomedical approaches inadequately capture Indigenous understandings of health, which are grounded in relationships and responsibilities rather than individual risk factors (22). Our findings add to the literature demonstrating that healthy families are foundational to the wellbeing and survival of Indigenous communities across the lifespan (23–25). Participants described pregnancy and parenting as communal and intergenerational processes shaped by restored family relationships, cultural continuity, identity, and supportive environments. These findings underscore the importance of family systems in perinatal wellbeing, reaching beyond nuclear family models to include grandparents, extended kin, and chosen family. This emphasis on broader kinship networks aligns with evidence showing kinship networks as protective systems that support caregiving, identity formation, and wellbeing (11, 17, 19, 22–25).

Participants' narratives reinforce evidence that intergenerational trauma and colonization fractured Indigenous kinship structures, parenting roles, and knowledge transmission (26, 27), negatively affecting family health. For some, this pattern was manifested through disrupted relationships and cycles of abuse. The RSHFS model highlights the importance of healing across multiple relational levels to support healthier perinatal experiences, while also emphasizing the resilience and agency of families striving for improved wellbeing.

Participants consistently framed culture and language as fundamental to identity and health, reinforcing research that positions cultural continuity as a determinant of wellbeing (28–30). Engagement in ceremony, land-based practices, and language learning was detailed as grounding, protective, and healing, particularly during pregnancy and early parenting. Similarly, cultural engagement has been shown to support the mental, emotional, and spiritual wellbeing of Indigenous parents and children (10, 12, 30). Participants also articulated how the loss of culture and language contributed to disconnection and poor health, reflecting broader colonial disruptions of Indigenous identity formation (31). At the same time, many expressed a strong desire to reclaim cultural knowledge and teachings, suggesting that perinatal programs may provide a critical opportunity for cultural resurgence.

Experiences of racism, stereotyping, and dismissal within health care settings, as described by some participants, mirrored those reported by Indigenous women in other maternity care settings (32, 33). This reinforces calls from the Truth and Reconciliation Commission of Canada (13) for systemic changes to improve cultural safety in health care. Accounts of respectful, relational care vs. harmful encounters identified in our study highlight the critical roles of health care provider attitudes, institutional policies aimed at equalizing structural power imbalances, and system flexibility (34). Research has shown that culturally safe care improves trust, engagement, and outcomes for Indigenous patients, particularly when Indigenous knowledge, Elders, and community priorities are meaningfully integrated (34, 35).

From a health promotion perspective, these findings emphasize that effective perinatal interventions must move beyond individual behavior change models and instead support culturally grounded, family-centered, and community-led approaches. The findings also highlight the importance of relational and cultural protective factors as key components of Indigenous health promotion and prevention strategies. Future research should continue to support community-led development and evaluation of perinatal programs, refine Indigenous-specific wellbeing indicators, and explore how family-centered models can be adapted across diverse Indigenous contexts while respecting distinctions-based approaches.

There are, however, several limitations to this study. Each Indigenous community has distinct histories, languages, cultural practices, and priorities; therefore, our findings cannot be generalized broadly. This study was conducted during the COVID-19 pandemic, and the number of survey respondents was relatively small. Additional efforts are needed to reduce barriers to participation and ensure information gathering remains relevant and accessible to communities. Furthermore, although the RSHFS model is deeply rooted in nêhiyawêwin (Cree language), data generation occurred primarily in English. Consequently, some spiritual or cultural nuances inherent in Cree concepts may have been “lost in translation” during the analysis. Finally, because this study was cross-sectional, it cannot determine whether the desires for healing or language learning translate into long-term behavioral or clinical health outcomes. However, as this research represents phase two of an ongoing partnership, future longitudinal work will aim to capture these changes throughout the life course of the children and families involved.

### 4.1 Conclusion

The RSHFS model resonated strongly with participants and encompassed domains viewed as important to wellbeing during childbearing age in Maskwacîs. By situating perinatal wellbeing within restored family systems, cultural continuity, and culturally safe environments, this study advances Indigenous health scholarship that challenges biomedical and deficit-based paradigms. Community-led approaches grounded in Indigenous knowledge offer important pathways for health promotion by supporting healing intergenerational trauma, strengthening identity, and supporting Indigenous families during pregnancy and early parenting.

## Data Availability

The datasets presented in this article are not readily available because this study adheres to the principles of Ownership, Control, Access, and Privacy (OCAP™) and will not make the interview data publicly available. Requests to access the datasets should be directed to roster@ualberta.ca.
