# Supplementary material for: Perinatal wellbeing through restoring healthy family systems in a nêhiyaw (Plains Cree) community: a mixed methods study
Source: Front Public Health. 2026 Jun 29;14:1823280. doi: 10.3389/fpubh.2026.1823280 (PMC13357864; doi:10.3389/fpubh.2026.1823280)
Supplement: Supplementary file 1 [file Table_1.docx]

**Supplementary Material**

**Positionality statements of authors**

RTO is a man of mixed European descent, including Danish, Scottish, German, Austrian, and Ukrainian ancestries. His Cree ceremonial name is Wâpastim (White Horse). Since birth, he has called Edmonton, Alberta home. His family has lived in this area for four generations and he continues to live there with his wife (who is of Cantonese Chinese descent) to raise his two teenage children. He positions himself as an equitable partner who supports strengths-based, solution-oriented, and community-driven approaches to research, building specifically on Indigenous ways of knowing. At the time of writing this manuscript RTO is the Indigenous Wellness Core of Primary Care Alberta scientific director, an adjunct assistant professor at the Universities of Alberta and Calgary, and an academic member of the Wâhkohtowin Research Group.

LL is a member of Neyaskweyahk, Ermineskin Cree Nation, in Maskwacîs, Alberta, located on Treaty 6 Territory. She has been involved in community-based research with and for her community since 2016. The projects she has been involved with include working with mature women’s wellness and aging, intergenerational cohesion, Indigenous gender and wellness, pregnancy, maternal health, and rebuilding healthy family structures. At the time of writing this manuscript LL is a community researcher with the Wâhkohtowin Research Group.

DL is from, and lives, in Neyaskweyahk, Ermineskin Cree Nation, part of Maskwacîs, Treaty 6 Territory in Alberta. She is a mother of four and is a former community researcher with the Wâhkohtowin Research Group. With a science and Nêhiyaw (Cree) background, she enjoys contributing part of her time to changing the way we view research and promoting Indigenous healthy family systems. At the time of writing, DL is an after-degree student and research assistant with the Faculty of Nursing at the University of Alberta.

MS is from Samson Cree Nation in Maskwacîs. She is proud to be a part of a research group that has given her the opportunity to play a part in improving the health and well-being of her own home community and allow families the opportunity to learn Cree traditional teachings and language. At the time of writing, MS has a community engagement and administrative support role with the Wâhkohtowin Research Group.

PL’s Cree name is Pisimawasis (Sunchild), from Maskwacîs, Treaty 6 Territory. He is of Cree and Anishinaabe descent, with a traditional knowledge and upbringing in the Nêhiyaw (Cree) way of life. He aims to raise his family in the same way he was taught. He is currently the coordinator for the Deadly Dads’ Support Society and a community researcher with the Wâhkohtowin Research Group.

LB is from Treaty 6 and a member of the Pelican Lake First Nations. At an early age, her maternal grandmother instilled a healing foundation entirely within the Cree language, leading to a solid 30-year career as a registered nurse. This grounding embedded a rigorous language-based research practice, to inform Indigenous-led cancer research. LB is the chief executive officer of Alberta First Nations Information Governance Centre and was elected president of the Canadian Indigenous Nurses Association in 2019. LB is a leader in positioning Knowledge Holders/Practitioners as rights holders to guide community-based research.

WCH is a first-generation Chinese Canadian who was born on Treaty 6 Territory with ancestral ties to the Guangdong Province of China. She has over two decades of experience in education, social service, and not-for-profit sectors as an educator, director, coresearcher, community builder, and evaluator. She is committed to making visible the invisible realities of marginalized and vulnerable populations to influence social change. She is a former academic member of the Wâhkohtowin Research Group. At the time of writing, WCH is a community development consultant.

MG is a white settler from Treaty 1 Territory—homeland of the Red River Métis (Manitoba). Tkaronto (Toronto) has been her home for the last 10 years. MG has worked in partnership with Indigenous women, girls, and gender-diverse people through a variety of roles since 2008 in Alberta, Manitoba, and Ontario. At the time of writing, MG is an assistant professor at Trent University.

JRB is a white cis woman born and raised in amiskwacîwâskahikan (Edmonton, Alberta),

located on Treaty 6 Territory and the Métis North Saskatchewan River Territory. She is of French Canadian, Italian, and Dutch ancestry maternally, and French Canadian and Irish Scot ancestry paternally. JRB has supported strengths-based, community-led, and mixed methods research on topics including perinatal health, ethnoarchaeology, and advocacy and activism in forensic anthropology. At the time of writing, JRB is an academic member and research coordinator for the Wâhkohtowin Research Group.

RCB is a white woman of mixed Western European descent, including Scottish, English, and German.  She was born and raised in the Great Lakes area of Canada and the USA and moved to Treaty 6 Territory. She has lived and worked on Treaty 6 land, in the area called amiskwacîwâskahikan (Edmonton, Alberta), for close to 30 years. At the time of writing, RCB is a professor emeritus with the University of Alberta and an academic member of the Wâhkohtowin Research Group.
